# Obstetric Admissions in ICU in a Tertiary Care Center: A 5-Years Retrospective Study

**DOI:** 10.5005/jp-journals-10071-23163

**Published:** 2019-05

**Authors:** Maria Vargas, Annachiara Marra, Pasquale Buonanno, Carmine Iacovazzo, Vincenzo Schiavone, Giuseppe Servillo

**Affiliations:** 1-4,6 Department of Neurosciences, Reproductive and Odontostomatological Sciences, University of Naples “Federico II”, Naples, Italy; 5 Department of Anesthesia and Intensive Care, Pineta Grande Clinic, Caserta, Italy

**Keywords:** Intensive care unit, Obstetric patient, Postpartum patient, Tertiary care facility

## Abstract

**Background and Aim:**

Obstetric patients admitted to intensive care unit (ICU) represent a challenge to physicians. The purpose of this study is to evaluate the incidence, characteristics, and mortality of pregnant and postpartum patients requiring ICU admission.

**Materials and Methods:**

A retrospective cohort study was performed between January 2008 and December 2013 at the University Hospital Federico II of Naples including pregnant and puerperal women until the 42nd day of postpartum and admitted to ICU.

**Results:**

Patients admitted with an obstetric diagnosis had a higher incidence of at least one previous cesarean section, were treated more with hysterectomy, had an increasing incidence of hemodynamic instability, had more postpartum admission, had a higher TISS-28 score, and required more endotracheal intubation than patients admitted with non-obstetrics diagnosis.

**Conclusion:**

A shared approach including a close collaboration between ICU and obstetric ward may be useful to reduce ICU admission and to improve maternal and foetal outcomes.

**How to cite this article:**

Vargas M, Marra A, Buonanno P, Iacovazzo C, Schiavone V, Servillo G. Obstetric Admissions in ICU in a Tertiary Care Center. A 5-years Retrospective Study. Indian J Crit Care Med 2019;23(5):213–219.

## INTRODUCTION

Obstetric patients admitted to intensive care unit (ICU) represent a challenge to intensivists due to the physiological adaptations and progress of disease during pregnancy and puerperium. The challenge is even more difficult because of the necessity to safeguard health of the mother and survival of the foetus. There is a striking connection between the number of maternal deaths and the accessibility to ICU care since the countries with the highest number of maternal deaths are also those with the lowest number of beds per capita in ICU. The injury severity scores at ICU admission in developing countries are significantly higher compared to developed countries.^1^ This means that the delay of accessibility to ICU care is the leading factor for increasing maternal mortality. The prevalence of ICU admissions among obstetric patients varies from 1 to 9 for 1,000 pregnancies, representing less than 1% of all ICU admissions.^2,3^ In the United Kingdom and United States, the ICU admission rate is 0.9% of all pregnancies with mortality rates ranging from 5 to 20%.^4^ In a retrospective analysis of 1,023 critically ill obstetric patients,^5^ the authors identified age, race, socio-economic conditions, and prenatal care as risk factors for ICU admission. Reasons for ICU admissions included conditions related to pregnancy (preeclampsia-eclampsia, haemorrhage, cardiomyopathy, puerperal infections, etc.), conditions unrelated (congenital and valvular heart disease, pulmonary hypertension, renal failure, etc.) to pregnancy, and medical pathologies worsened by pregnancy.^6^ Obstetric patients admitted in ICU are younger and have less comorbidities than general female population.^7^ Obstetric ICU patients also have lower mortality rate compared with general ICU female population (2–3% vs 20%).^7^ The aim of the present study is to evaluate the incidence, characteristics, and mortality of pregnant and postpartum patients requiring ICU admission in a tertiary care facility.

## MATERIALS AND METHODS

This retrospective cohort study was performed at the University Hospital Federico II of Naples, Italy, a tertiary care facility with 2,300 births per year and a referral centre for high risk pregnancies. The institutional ethics committee approved the study protocol. This hospital is equipped with an obstetrical emergency room with dedicated staff and has 14 beds in general ICU.

In this study, we included all pregnant and puerperal women until the 42nd day after the birth, aged more than 18 years, and admitted to the ICU between 1st January 2008 and 31st December 2013. The obstetric patients were identified from the ICU admission records; the readmissions occurred within 30 days have been only counted once. Characteristics of the patients admitted in ICU were collected from the medical charts available in the archive of our department, recorded on a pre-filled form, and entered in a computerized database using MS Office Excel 2007 (Microsoft, Redmond, WA, USA).

Reasons of ICU admission were classified in two groups according to presence of disease related (obstetric group) or unrelated (non-obstetric group) to pregnancy.

For disease related to pregnancy, we included the following conditions:

Severe pre-eclampsia was identified by the presence of arterial hypertension (>160/110 mm Hg with proteinuria higher than 2 gm over 24 hours), with one or more of the following signs or symptoms like oliguria, epigastric pain, migraine, blurry sight, and pulmonary oedema.

Eclampsia was defined as the onset of seizures during pregnancy or in the early postpartum period not due to drugs or other maternal diseases.

HELLP (hemolysis, elevated liver enzymes, low platelets) syndrome was characterized by haemolysis (bilirubin> 1 mg/dL or haptoglobin <0.5 mg/dL or schistocytes in the peripheral blood), low platelet counts (<100,000/mm^3^), and increase of hepatic cytolysis indexes (Alanina Amino Transferase >70 U/L or Gamma-Glutamyl Transferase >70 U/L).

Major obstetric hemorrhage was defined as blood loss between 1000 mL and 1500 mL in a short period or more than 2500 ml in 24 hours.

Massive obstetric hemorrhage was defined as a blood loss higher than 1500 mL, or the need for blood transfusions of more than 4 units of packed blood cells, or surgery for the control of hemostasis (embolization or hysterectomy), or hemoglobin level lower than 4 g/dL or presence of signs of shock.^8,9^

Peripartum cardiomyopathy was identified as the development of left ventricular dysfunction (ejection fraction <45%) in the last month of pregnancy or within the first 5 months during the postpartum period, in the absence of preexisting cardiac diseases and any identifiable causes of congestive heart failure.^10^

For non-obstetric admission, we included patients who need admission in ICU for reasons not related to pregnancy.

At the ICU admission for each patient, we measured the sequential organ failure assessment (SOFA) score,^11^ simplified acute physiology score II version (SAPS II) score,^12^ ICU length of stay (ILOS), hospital length of stay (HLOS), and therapeutic intervention scoring system (TISS 28).^13^

Categorical data were expressed in percentages and compared with the chi-square test. Continuous data were reported as mean and standard deviation and compared with the Student's t-test for unpaired samples. Statistical significance was set with a *p* value less than 0.05. The statistical analysis was carried out using the IBM SPSS software (version 20.0, IBM Corporation, New York, USA).

## RESULTS

From 1st January 2008 to 31st December 2013, 66 obstetric patients were admitted to our ICU, representing 0.5% of hospital deliveries (66/13 422 deliveries) and 2.9% of all ICU admissions (66/2 287). The yearly percentage of obstetric patients compared to all patients admitted to ICU ranges from 1.6 to 4.2% (2008: 3.4%; 2009: 4.2; 2010: 1.6%; 2011: 3.5%; 2012: 3.2%; 2013: 1.6%). Only one woman died in 2010. Table 1 shows the maternal characteristics and comorbidities. At the ICU admission, 38 patients (57%) had a diagnosis related to obstetric disease while 28 (43%) had a non-obstetric related diagnosis. The incidence of previous cesarean section was statistically different between the considered groups (obstetric patients: 22/38; non-obstetric: 6/28; *p* = 0.006). During the study period, more than 90% of patients in each group had a cesarean section. Hysterectomy was performed in 45% of obstetric patients with statistical significance (*p* = 0.000). Table 2 shows the mode of delivery and related anaesthesiology procedures.

**Table 1:** Maternal characteristics and comorbidities

	*Total* *n = 66* *(mean; SD; range)*	*Obstetric ddmissions* *n = 38* *(mean; SD; range)*	*Non obstetric admissions* *n = 28* *(mean; SD; range)*	*p value*
Maternal age (y)	31.8; 6.7; 18–44	32.9; 6.3; 18–44	30.2; 7; 19–43	0.095
Body mass index (kg/m^2^)	28.9; 5.3; 21.3–50.5	28.8; 4.2; 22.3–36.4	28.8; 6.6; 21.3–50.5	0.760
Gestational age (y)	33; 6; 35–37	34; 4; 25–40	30; 8, 4–40	0.098
First pregnancy	22; 33%	12; 31%	10; 36%	0.928
Patients with at least one previous cesarean section	28; 43%	22; 58%	6; 21%	0.006
Multiple pregnancy^**^	5; 7%	2;0,05%	3; 0,1%	0.721
*Comorbidities*	*Total n = 66* *(n; %)*	*Obstetric admissions n = 38* *(n; %)*	*Medical admission n = 28* *(n; %)*	*p value*
Heart disease	10; 15%	5; 13%	5; 18%	0.858
Arterial hypertension	2; 3%	2; 3%	0	0.612
Hemostatic disorder	5; 8%	1; 5%	4; 14%	0.148
Morbid obesity (BMI >35)	2; 3%	2; 5%	0	0.612
Asthma	2; 3%	1; 2.5%	1; 3.5%	0.612
Autoimmune disease	2; 3%	1; 2.5%	1; 3.5%	0.612
Hypothyroidism	4; 6%	3; 8%	1; 3.5%	0.467
Malignancy	3; 4%	2; 5%	1; 3.5%	0.744
Chronic renal failure	1; 1%	0	1; 3.5%	0.876
Diabetes mellitus type I	1; 1%	1; 2.5%	0	0.876
Chronic infection (HIV, HBV)	2; 3%	1; 2.5%	1; 3.5%	0.61
Cerebrovascular accident	2; 3%	2; 5%	0	0.612
Others^****^	6; 9%	4; 10%	2; 7%	0.964

^**^4 twin and one triplet pregnancies;

^****^Systemic mastocytosis, chronic venous insufficiency, chronic inflammatory demyelinating polyneuropathy, kidney stone, cerebral angiomatosis, cushing syndrome

**Table 2:** Mode of delivery and anesthesiological procedures

	*Total* *n = 63* *n; %*	*Obstetric diagnosis* *n = 38* *n; %*	*Non obstetric diagnosis* *n = 25* *n; %*	*p value*
Vaginal delivery	3; 5%	2; 5%	1; 4%	0.708
Cesarean section^^*^^ – Elective – Planned – Urgent – Emergent	60; 95% 11 13 29 7	36; 95% 3 12 16 5	24; 96% 8 1 13 2	0.708
Spinal/epidural anesthesia	24; 40%	14; 37%	10; 40%	1
General anesthesia	36; 60%	22; 63%	14; 60%	0.912
ASA I-II	22; 37%	12; 32%	10; 40%	0.677
ASA III-IV	36; 60%	26;68%	13; 52%	0.294
ASA V	2; 3%	0	2; 8%	0.299
Hysterectomy	17; 27%	17; 45%	0	0.000
Uterine artery embolization	3; 5%	3; 8%	0	0.403

^*^According to Lucas classification of urgency of cesarean section

**Table 3:** Intensive care unit admission diagnosis

*Obstetric Diagnosis*	*38/66 (58%)*	*Non Obstetric Diagnosis*	*28/66 (42%)*
Hypertensive disease HELLP syndrome^*^ Eclampsia Preeclampsia AFLP^**^	16/66(25%) 7/16 4/16 3/16 2/16	Non obstetric sepsis Pneumonia ^*****^ Pyelonephritis Other (H1N1) Pulmonary embolism	5/66 (7%) 3/5 1/5 1/5 5/66 (7%)
Major hemorrhage Uterine atony Abnormal invasive placenta Retained placental tissue Abruptio placentae DIC/Intrauterine fetal death^***^ Puerperal sepsis Peripartum cardiomyopathy	20 (31%) 6/20 9/20 1/20 2/20 2/20 1 (1%) 1 (1%)	Heart diseases Acute thrombocytopenia Acute renal failure Pulmonary atelectasis Miscellaneous Major trauma Seizures Bowel obstruction Chronic inflammatory demyelinating polyneuropathy Oral cancer Systemic mastocytosis Pulmonary edema	4/66 (6%) 3/66 (5%) 2/66 (3%) 2/66 (3%) 7/66 (11%) 1/7 1/7 1/7 1/7 1/7 1/7 1/7

^*^HELLP: Hemolysis, elevated liver enzymes, low platelets;

^**^AFLP: Acute fatty liver of pregnancy;

^***^DIC: Disseminated intravascular coagulation;

^****^Two cases of community acquired pneumonia (CAP, *Streptococcus pneumonia*) and one case of hospital acquired pneumonia (HAP, *Pseudomonas aeruginosa*)

Table 3 shows the reasons for ICU admission. The most frequent reason of ICU admission in obstetric group was major haemorrhages (31%) followed by hypertensive disorders (25%). The most frequent reason of ICU admission in non-obstetric group was pulmonary embolism (7%) and non-obstetric sepsis (7%).

Table 4 shows the incidence of organ failures at the ICU admission. Hemodynamic failure was the main reason of ICU admission in obstetric group (*p* = 0.011). Hemodynamic instability included severe arterial hypertension resistant to pharmacological treatment with two or more drugs (3/27), cardiac dysfunction (3/27), and severe sepsis/septic shock (2/27). Hemodynamic failure was more frequent in obstetric group (*p* = 0.011). Respiratory failure was more frequent in the non-obstetric group (43%) (*p* = 0.000). Two patients showed cardiorespiratory failure due to massive pulmonary thromboembolism. For post-operative monitoring, 18% of non-obstetric patients with preexisting pathologies were admitted in ICU (*p* = 0.012).

Table 5 shows characteristics and complications during the ICU stay. In non-obstetric group, 32% of patients were admitted before they gave birth while 100% of obstetric patients were admitted in the postpartum period. Nine patients out of 66 were admitted while still pregnant. Among these patients, four underwent emergency cesarean section (c-section), two patients gave birth after the ICU discharge, one patient had a spontaneous abortion, and two patients were lost to the follow-up. The most frequent complication during ICU stay was the need of surgery.

**Table 4:** Organ failures at the ICU admission

	Total n = 66 (n; %)	Obstetric admissions n = 38 (n; %)	Non obstetric admissions n = 28 (n; %)	p value
Hemodinamic failure	27; 41%	23; 61%	4; 14%	0.011
Respiratory failure	12; 18%	0	12; 43%	0.000
Hemodinamic and respiratory failure	2; 3%	1; 3%	1; 4%	0.831
DIC/TTP/HUS^*^	9; 14%	8; 21%	1; 4%	0.070
Neurological dysfunction	6; 9%	4; 11%	2; 7%	0.665
Hepatic failure	2; 3%	2; 5%	0	0.229
Acute kidney failure	2; 3%	0	2; 7%	0.106
Bowel obstruction	1; 1%	0	1; 4%	0.248
Postoperative monitoring	5; 8%	0	5; 18%	0.012

^*^DIC: Disseminated intravascular coagulation; *TTP*: Thrombotic thrombocytopenic purpura; *HUS*: Hemolytic uremic syndrome

**Table 5:** Characteristics and complications of ICU stay

	*Total* *n = 66*	*Obstetric admissions* *n = 38*	*Non obstetric admissions* *n = 28*	*p value*
Antepartum admission (n; %)	9; 14%	0	9; 32%	0.000
Postpartum admission (n; %)	57; 86%	38; 100%	17; 78%	0.000
HLOS (days) (mean; SD; range)	18; 9; 1–50	20;9; 6–42	17; 10; 1–50	0.1793
ILOS (days) (mean; SD; range)	5; 4;1–29	5; 4; 2–23	4; 5;1–29	0.559
Endotracheal Intubation at admission (n;%)	35; 53%	25; 66%	10; 36%	0.031
SAPS II (mean; SD; range)	26; 13; 16–36	28;12; 8–64	23; 15; 3–51	0.155
TISS-24 (mean; SD; range)	30; 11; 5–59	34; 11; 16–59	27;12; 5–56	0.019
SOFA score (mean; SD; range)	4;3; 0–12	5;3; 1–12	4; 3; 0–11	0.051
*Complications*				
*Related to ICU admission diagnosis*	*Total n = 66 (n; %)*	*Obstetric admissions n = 38 (n; %)*	*Non obstetric admissions n = 28 (n; %)*	*p value*
Need for re-surgery	10;15%	5;13%	5;18%	0.858
PRES	5; 8%	5;13%	0	0.127
Acute renal failure	4; 6%	2; 5%	2; 7%	0.837
Cerebral ictus (ischemic, hemorrhagic)	3; 5%	3; 8%	0	0.332
DIC	2; 3%	2; 13%	0	0.612
Postoperative paralytic ileus	2: 3%	2;13%	0	0.612
Miscellaneous^*^	2; 3%	1; 3%	1; 2%	0.612
Related to ICU stay				
CVC related infection	2; 3%	1; 3%	1; 2%	0.612
VAP	2; 3%	1; 3%	1; 2%	0.612
Pulmonary edema due to volume overload	2; 3%	2; 13%	0	0.612

*HLOS:* Hospital length of stay; *ILOS:* ICU length of stay; *SAPS II:* Simplified acute physiology calculated on the worst values in the first 24 hours; *TISS-24:* Therapeutic intervention score system in the first 24 hour; *SOFA:* Severe organ failure assessment; *PRES:* Posterior reversible encephalopathy syndrome;

^*^Cerebral venous thrombosis and hepatorenal syndrome; *DIC:* Disseminated intravascular coagulation; *VAP:* Ventilatory associated pneumonia

SOFA and SAPS scores did not differ between the groups, while TISS score was higher in the obstetric patients (*p* = 0.019).

Table 6 shows the characteristics and outcome of patients according to previous c-section. In our study, a history of one or more previous c-sections was associated to a higher risk of major hemorrhages (*p* = 0.011), hysterectomy (*p* = 0.000), and need of packed red blood cells transfusions (*p* = 0.050).

Sixty-seven infants were born alive with a birth weight (mean, DS) of 2370 ± 900 gm (range 410–4200), at a gestational age (mean, DS) of 35 ± 4 weeks (range 25–40) and with an Apgar score at 5 minutes (mean, DS) of 8 ± 1 points (range 2–9). A new-born died few minutes after birth; there were 3 intrauterine foetal deaths and one spontaneous abortion. Perinatal mortality rate was 6%.

**Table 6:** History of previous cesarean section (CS) and outcome

	*Total* *n = 66*	*No previous cesarean section n = 38*	*One or more previous cesarean section n = 28*	*p value*
Non-obstetric admissions (n; %)	28; 42%	22; 48%	6; 21%	0.067
Obstetric admissions (n; %)	38; 57%	16; 42%	22; 79%	0.067
Major hemorrhage (n; %)	20; 30%	5; 13%	15; 54%	0.011
Abnormal invasive placenta (placenta accreta and/or percreta) (n; %)	9; 14%	1; 20%	8; 53%	0.436
Hysterectomy	17; 26%	1; 3%	16; 57%	0.000
HLOS (mean, SD, range)	28; 6; 1–55	19; 10; 1–50	18; 10; 7–42	0.988
ILOS (mean; SD; range)	37; 4; 1–27	5; 4; 1–29	4; 4; 2–23	0.798
SAPS II, (predicted mortality %) (mean; SD; range)	52; 15; 2–67	26; 13; 3–56	26; 14; 6–64	0.909
Patients receiving red blood cells transfusion; (n; %)	32; 48%	14; 37%	18; 61%	0.050
Patients receiving mechanical ventilation; (n; %)	45; 68%	24; 63%	21; 78%	0.451
Patients developing at least one complication during ICU stay (n; %)	21; 32%	14; 37%	7; 22%	0.451

## DISCUSSION

Maternal mortality reviews are globally used to assess the quality of healthcare services. In this retrospective study, we found that patients admitted in ICU with a disease related to pregnancy had a higher incidence of at least one previous cesarean section compared with patients with non-obstetric patients, 45% of patients in obstetric group were treated with hysterectomy compared with the patients in non-obstetric group, hemodynamic instability was higher in obstetric group while respiratory failure and postoperative monitoring were higher in non-obstetric group, patients with unrelated pregnancy diseases were admitted in ICU for postoperative monitoring, ICU admission before the birth was more frequent in non-obstetric group while postpartum ICU admission was more frequent in the obstetric group, TISS-28 was higher in obstetric group, and endotracheal intubation was more required in patients belonging to the obstetric group.

In the sub analysis for evaluating the outcome of ICU admission between patients with or without previous cesarean section, we found that patients with one or more previous c-section had a higher incidence of major hemorrhages, hysterectomy, and red blood cells transfusions. To our knowledge, this is the first study reporting the reasons of ICU admission in pregnant patients divided according to presence of diseases related (obstetric group) or unrelated (non-obstetric group) to pregnancy.

In line with the current literature, 79% of the study patients had a history of cesarean delivery.^14^

Obstetric ICU admission is a rare event in developed countries, although there are several potentially fatal conditions which may occur in peripartum period. In our study, approximately five women every 1,000 deliveries were admitted in ICU. International ICU admission rates varied from 0.7 to 1.3%, while 0.2–0.4% in developed countries.^15,16^ Also, the percentage of obstetric patients compared to the total of ICU admissions was comparable with the international data that ranged from 0.5 to 10.2%.^17^

In this study, patients admitted with pregnancy related diseases had a higher incidence of at least one previous cesarean section compared with patients without obstetric related diseases. Furthermore, 45% of patients in obstetric group were treated with hysterectomy compared with none of patients with non-obstetric group. As far as we know, no previous studies reported this relationship. Hysterectomy mainly occurred in postpartum period because of uterine atony.^6,7^ Hysterectomy, as a major surgical procedure, is associated with high complication rates.^6,7^ Patients treated with hysterectomy should be carefully monitored in postoperative period, probably this is the reason why we have a high incidence of hysterectomy in our obstetric group.

In this study, hemodynamic instability (41%), as a consequence of postpartum hemorrhage, was the most frequent reason of ICU admission followed by respiratory failure (31%) due to pneumonia and pulmonary atelectasis, severe coagulopathies (14%), and neurological dysfunctions (9%). Hemorrhagic shock was the most common indication for ICU admission.^17^ The indications for mechanical ventilation in obstetric patients have been not properly identified.^18,19^ The percentage of mechanical ventilated patients in this study was relatively high (68%) compared to the current literature.^17,18,20^ In this study, the most common indications leading to mechanical ventilation were the need of inotropes and vasopressors in the postoperative period, the severe respiratory failure, and neurological dysfunction with the impairment of the airway reflex. According to this, mechanical ventilation was mainly administered through the endotracheal tube, otherwise it was a non-invasive support. Current evidence-based ventilatory management has been developed from studies that excluded pregnant women. The uncertainty in management of obstetric patient relates not only to the ideal ventilator settings for these patients, but also to the optimal oxygen and CO_2_ targets, and whether to emulate the normal maternal respiratory alkalosis.^20^ However, the optimal setting of mechanical ventilation in the obstetric patients should take into account the physiological changes of pregnancy. Mechanical ventilation is the baseline treatment in the acute respiratory distress syndrome (ARDS). In severe ARDS, the current literature recommends to use lower tidal volume with lower inspiratory pressure^21^ while additional evidences are needed to recommend for or against the extracorporeal membrane oxygenation (ECMO).^21^ Published experience with ECMO in obstetric patients is still limited and associated with high incidence of hemorrhage and hypercoagulability.^22^

This study showed a 95% prevalence of cesarean section among the patients admitted in ICU. In 2000, an Italian study reported a 90.2% incidence of cesarean sections in critical obstetric patients.^23^ According to Lucas classification, cesarean section can be classified as emergent (immediate threat to mother and/or foetus life), urgent (compromised maternal conditions and/or of the foetus, which is not immediately life-threatening), planned (needing early delivery but no maternal or foetal compromise) or elective.^24^ In this study, 22% of c-sections were planned (nine for abnormal invasive placenta, two for malignant metastatic cancer, two for valvular cardiomyopathy, and one for HIV), 61% were emergent or urgent, while only 17% could be classified as elective.

Hemorrhage was the most frequent reason of ICU admission in obstetric group.^14^ Multiple parity, HELLP syndrome, disseminated intravascular coagulation (DIC), and cesarean section were the risk factors for obstetric hemorrhage. In our study, 49% of the patients received packed red blood cells transfusions, 33% received fresh frozen plasma, 10% received platelets, and 3% received clotting factor concentrates. Sixteen patients (24%) underwent hysterectomy immediately after the cesarean section or within the 2 days after the birth and three patients underwent embolization of uterine arteries. The most frequent condition associated with hysterectomy was abnormal invasive placenta followed by uterine atony. In one case, hysterectomy was performed because of puerperal sepsis due to endometritis unresponsive to antibiotics administration.

According to a study of the Italian National Health System Institute^25^ in six Italian regions during the period 2004–2005, obstetric hemorrhage was most frequently caused by placental abruption, followed by placenta previa. In this study, the greater prevalence of abnormal placentation could be due to the elevated number of c-sections performed in Italy (60% vs the national average of 38%), being the cesarean section itself one of the principal risk factors for abnormal placental insertion. On the other hand, ultrasound and MRI scans currently allow diagnosis of the anomalies of placentation before the birth.

C-section is an independent risk factor for severe maternal morbidity and mortality. In a large study, Silver et al. showed that the risk of severe complications considerably increases with the number of previous cesarean sections,^26^ other authors did not confirm this conclusion.^27^ In this study, the presence of one or more previous c-sections increased the probabilities of severe obstetric hemorrhages, packed red blood cells transfusions, and emergency hysterectomy, but they did not influence the length of ICU stay, the onset of complications, and the need for mechanical ventilation. Multiple parity, presence of HELLP syndrome, presence of DIC, and the performance of a cesarean delivery appeared as the precipitating factors for severe hemorrhages in obstetric patients.^28^

The mean length of ICU stay was 5 days, without any significant difference between patients with obstetric or non-obstetric diagnosis; whereas, 54% of the patients stayed in ICU for three days or less. This result fitted with the other studies^29^ and reflected the transitory nature of the majority of obstetric pathologies fixed by the delivery and the placental expulsion.

The incidence of maternal deaths in developed countries is about 2–3/100 deliveries (range 0–12%), and in developing countries, it is 10–25%.^18,29^ We observed only one maternal death because of massive pulmonary embolism, corresponding to a mortality rate of 1.5%, despite a predicted mortality rate of 12%. Regarding the causes of maternal deaths in developed countries, Homer et al. reported the embolism of amniotic fluid in 28% of cases, both the pregnancy hypertensive disorders and venous thromboembolism in 17% of cases, and obstetric haemorrhage in 14% of patients.^30^ The Eight Report of National Enquiries into Maternal Death in UK showed that up to 50% of maternal deaths are due to medical preexisting diseases exacerbated by pregnancy.^4^

The ICU prognostic scoring systems, such as APACHE II, does not take into account the physiological changes that occur in peripartum period nor the pathophysiological peculiarity of pregnancy related diseases. The SAPS II score is more reliable than the APACHE II to predict mortality in obstetric patients^28,31,32^ and may help to identify high-risk patients. In our study, the SAPS II score and the SOFA score seemed to be useful to predict ICU length of stay, but not the mortality rate (duration of stays >72 hours: average SAPS II score of 34±12 and average SOFA score of 6±3; stays <72 hours: SAPS II score 19±11 and SOFA score 3±2; *p* <0.05). A modified early obstetric warning system (MEOWS) chart is used from 20 weeks of gestation, when the woman is admitted to maternity wards, to the postnatal period up to the 6th week following childbirth. MEOWS score considers different physiological parameters specifically relevant for obstetric patients to recognize a possible deterioration in women's condition.^33^ MEOWS should be used in the obstetric ward as a warning or screening tool for women at risk of developing serious illness.

One of the main limitations of this study was the retrospective design. The low number of patients did not allow the identification of prognostic factors between survivors and non-survivors. Similarly, we were not able to identify risk factors for ICU admissions before the birth due to the lack of a control group.

## CONCLUSION

Management of the peripartum patients is a challenging aspect of critical care that requires consideration of the physiological changes associated with pregnancy and the well-being of the fetus. A shared approach including a close collaboration between ICU and obstetric ward may be useful to reduce ICU admission and to improve maternal and fetal outcomes.

## References

[1] Sultan P,, Arulkumaran, N,, Rhodes A. (2013;). Provision of critical care services for the obstetric population.. Best Pract Res Clin Obstet Gynaecol..

[2] Soubra SH,, Guntupalli KK. (2005:). Critical illness in pregnancy: an overview.. Crit Care Med..

[3] Kelsey JJ,, Pharm D. (2010;). Obstetric emergency in the ICU.. Crit Urgent Care PSAP VII..

[4] Cantwell R,, Clutton-Brock T,, Cooper G,, Dawson A,, Drife J,, Garrod D, (2011;). Saving mothers’ lives: reviewing maternal deaths to make motherhood safer 2006-2008. The eighth report of the confidential enquiries into maternal deaths in the United Kingdom.. BJOG..

[5] Panchal S,, Arria AM,, Harris AP. (2000;). Intensive care utilization during hospital admission for delivery.. Anesthesiology..

[6] Mahutte NG,, Murphy-kaulbeck L,, Le Q,, Solomon J,, Benjamin A,, Boyd ME. (1999;). Obstetric admissions to the intensive care unit.. Obstetr Gynecol..

[7] Price LC,, Germain S,, Wyncoll D,, Nelson-Piercy C. (2009;). Management of the critically ill obstetric patient.. Obstet Gynaecol Reprod Med..

[8] American College of Obstetricians and Gynecologists. (2006;). ACOG Practice Bullettin: clinical management guidelines for obstetrician-gynecologists number 76, October 2006: postpartum hemorrhage.. Obstet Gynecol..

[9] World Health Organization. (2009.). WHO guidelines for the management of the postpartum hemorrhage and retained placenta..

[10] Pearson GD,, Veille JC,, Rahimtoola S,, Hsia J,, Oakley CM,, Hosenpud JD (2000;). Peripartum cardiomyopathy: national heart, lung, and blood institute and office of rare diseases (National Institutes of Health) workshop recommendations and review.. JAMA..

[11] Vincent JL,, Mendonca A,, Cantraine F,, Moreno R,, Takala J,, Suter PM (1998;). Use of the SOFA score to assess the incidence of organ dysfunction/failure in intensive care units: results of a multicenter, prospective study.. Crit Care Med..

[12] Le Gall JR,, Lemeshow S,, Saulnier. (1993;). A new simplified acute physiology score (SAPS II) based on a European/North American multicenter study.. JAMA..

[13] Miranda DR,, de Rijk A,, Schaufeli W. (1996;). Simplified therapeutic intervention scoring system: the TISS 28 items result from a multicenter study.. Crit Care Med..

[14] Karnad DR,, Lapsia V,, Krishnan A,, Salvi VS. (2004;). Prognostic factors in obstetric patients admitted to an Indian intensive care unit.. Crit Care Med..

[15] Pollock W1,, Rose L,, Dennis CL. (2010;). Pregnant and postpartum admissions to the intensive care unit: a systematic review.. Intensive Care Med..

[16] Zeeman GG. (2006;). Obstetric critical care: a blueprint for improved outcomes.. Crit Care Med..

[17] Oliveira Neto AF,, Parpinelli MA,, Cecatti JG,, Souza JP,, Sousa MH. (2009;). Factors associated with maternal death in women admitted to an intensive care unit with severe maternal morbidity.. Int J Gynaecol Obstet..

[18] Cohen J,, Singer P,, Kogan A,, Hod M,, Bar J. (2000;). Course and outcome of obstetric patients in a general intensive care unit.. Acta Obstet Gynecol Scand..

[19] Jenkins TM,, Troiano NH,, Graves CR,, Baird SM,, Boehm FH. (2003;). Mechanical ventilation in an obstetric population: characteristics and delivery rates.. Am J Obstet Gynecol..

[20] Selo-Ojeme DO,, Omosaiye M,, Battacharjee P,, Kadir PA. (2005;). Risk factors for obstetric admissions to the intensive care unit in a tertiary hospital: a case-control study.. Arch Gynecl Obstet..

[21] Howell MD,, Davis AM. (2018;). Management of ARDS in adults.. JAMA.

[22] Agerstrand CA,, Abrams D,, Biscotti M,, Moroz L,, Rosenzweig EB,, D'Alton M (2016;). Extracorporeal membrane oxygenation for cardiopulmonary failure during pregnancy and postpartum.. An Thorac Surg..

[23] Loverro G,, Pansini V,, Greco P,, Vimercati A,, Parisi AM,, Selvaggi L. (2001;). Indications and outcome for intensive care unit admission during puerperium.. Arch Gynecol Obstet..

[24] Istituto Superiore di Sanità. ((2012)). Taglio cesareo. Una scelta appropriata e consapevole. Seconda parte..

[25] Senatore S,, Donati S,, Andreozzi S. (2012). Studio delle cause di mortalità e morbosità materna e messa a punto dei modelli di sorveglianza della mortalità materna..

[26] Silver RM,, Landon MB,, Rouse DJ,, Leveno KJ,, Spong CY,, Thom EA, (2005;). Maternal morbidity associated with multiple repeat cesarean deliveries.. Obstet Gynecol..

[27] Pallasmaa N,, Ekblad U,, Aitokallio-Tallberg A,, Uotila J,, Raudaskoski T,, Ulander VM, (2010;). Cesarean delivery in Finland: maternal complications and obstetric risk factors.. Acta Obstet Gynecol Scand..

[28] Togal T,, Yucel N,, Gedik E,, Gulhas N,, Toprak HI,, Ersoy MO. (2010;). Obstetric admissions to the intensive care unit in a tertiary referral hospital.. J Crit Care..

[29] Vasquez DN,, Estenssoro E,, Canales HS,, Reina R,, Saenz MG,, Das Neves AV, (2007;). Clinical characteristics and outcomes of obstetric patients requiring ICU admission.. Chest..

[30] Homer C,, Clements V,, McDonnell N,, Peek M,, Sullivan E. (2009;). Maternal mortality: what can we learn from stories of postpartum hemorrhage?. Women Birth..

[31] Karnad D,, Guntupalli KK. (2005;). Neurologic disorders in pregnancy.. Crit Care Med..

[32] Tempe A,, Wadhwa L,, Gupta S,, Bansai S,, Satyanarayana L. (2007;). Prediction of mortality and morbidity by simplified acute physiology score II in obstetric intensive care unit admissions.. Indian J Med Sci..

[33] Edwards SE,, Grobman WA,, Lappen JR,, Winter C,, Fox R,, Lenguerrand E (2015;). Modified obstetric early warning scoring systems (MOEWS): validating the diagnostic performance for severe sepsis in women with chorioamnionitis.. Am J Obstet Gynecol..

